# Supplementary figures and images for: Isolation and Characterization of a Novel Temperate Escherichia coli Bacteriophage, Kapi1, Which Modifies the O-Antigen and Contributes to the Competitiveness of Its Host during Colonization of the Murine Gastrointestinal Tract
Source: mBio. 2022 Jan 25;13(1):e02085-21. doi: 10.1128/mbio.02085-21 (PMC8787464; doi:10.1128/mbio.02085-21)

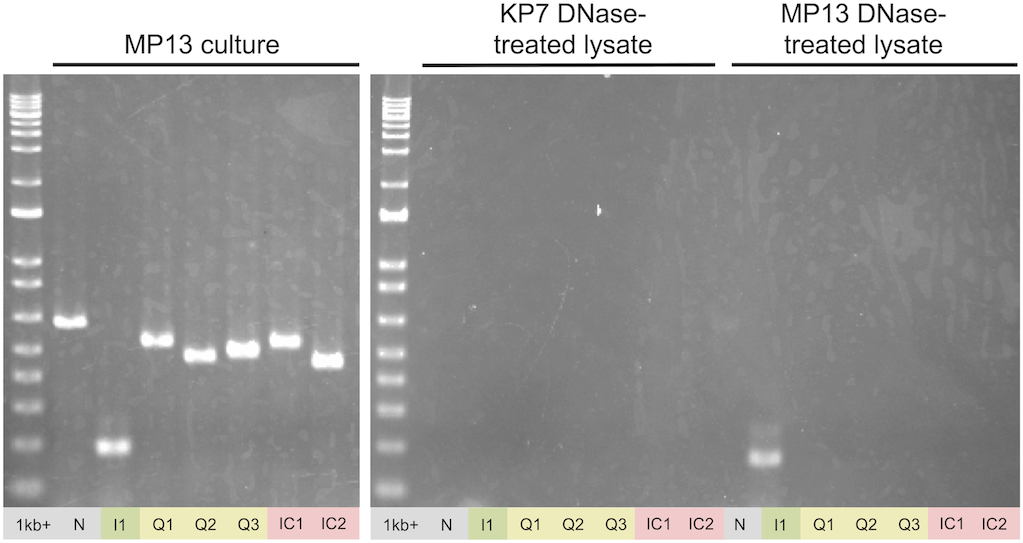

Supplement: FIG S1 [file mbio.02085-21-sf001.tif]

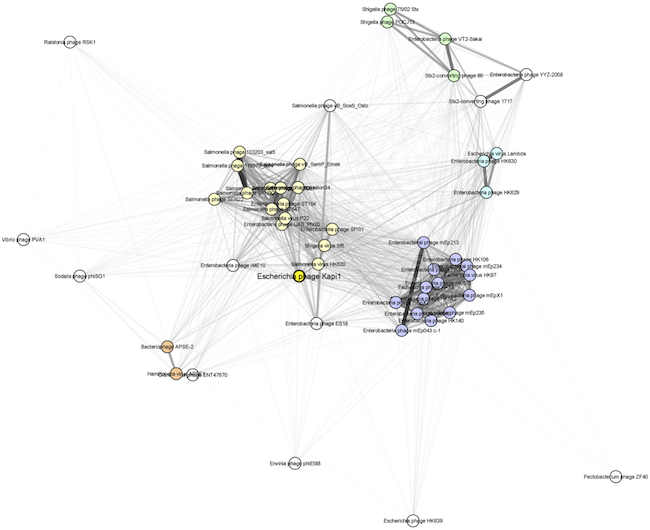

Supplement: FIG S2 [file mbio.02085-21-sf002.tif]

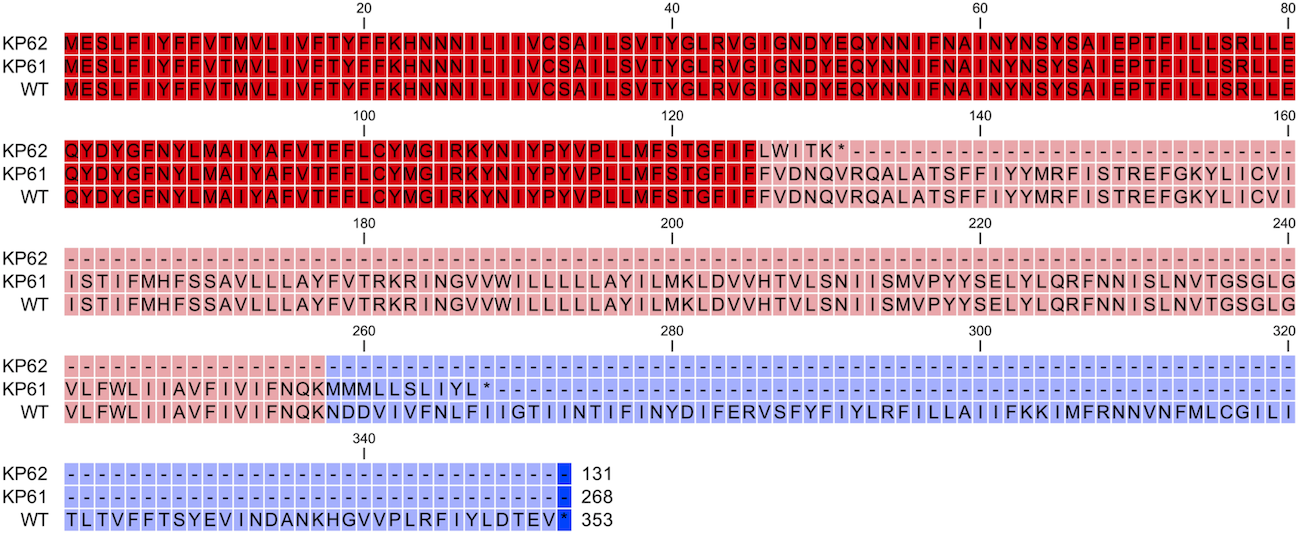

Supplement: FIG S3 [file mbio.02085-21-sf003.tif]

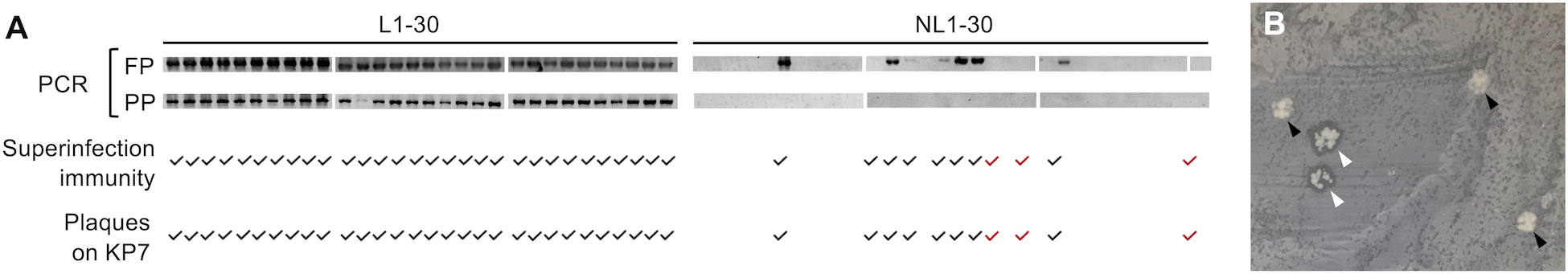

Supplement: FIG S5 [file mbio.02085-21-sf005.tif]

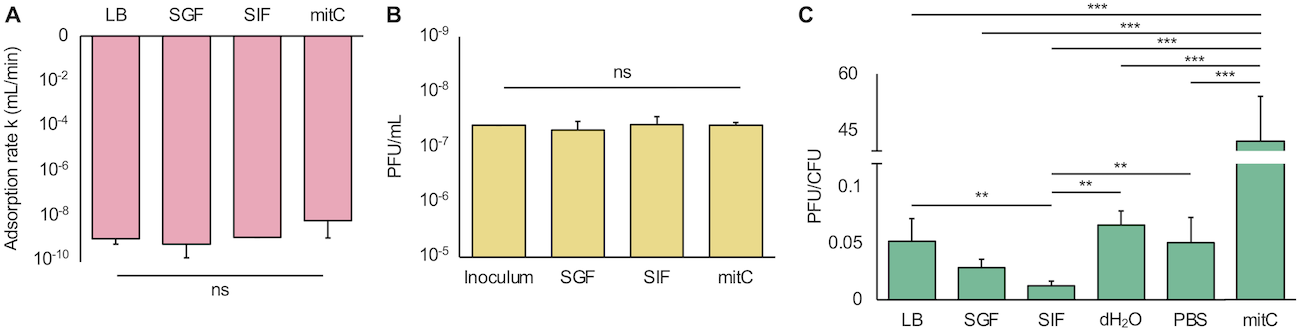

Supplement: FIG S4 [file mbio.02085-21-sf004.tif]
