# Supplementary material for: Isolation and Characterization of a Novel Temperate Escherichia coli Bacteriophage, Kapi1, Which Modifies the O-Antigen and Contributes to the Competitiveness of Its Host during Colonization of the Murine Gastrointestinal Tract
Source: mBio. 2022 Jan 25;13(1):e02085-21. doi: 10.1128/mbio.02085-21 (PMC8787464; doi:10.1128/mbio.02085-21)
Supplement: TABLE S1 [file mbio.02085-21-st001.docx]

**Table S1. Bacterial strains used in this study.**

| Strain | Description/Genotype | Source/Reference |
| --- | --- | --- |
| MP1 | *E. coli* isolated from feces of healthy laboratory mouse | (11) |
| MP7 | MP1 att_λ_::pML8 | (11) |
| KP7 | MP7 derivative lacking the Kapi1 prophage | This study |
| MP13 | MP1 att_λ_::pAS07 | (11) |
| KP61 | KP7 spontaneous mutant resistant to infection by Kapi1 | This study |
| KP62 | KP7 spontaneous mutant resistant to infection by Kapi1 | This study |
| KP81 | KP7 ∆*waaF::kan* | This study |
| KP120 | KP7 ∆*waaL::kan* | This study |
| KP121 | KP7 *attB::kan* | This study |
| KP158 | KP7 ∆*attB::kan* | This study |
| KP164 | MP13 ∆*recA::kan* | This study |
| Top10 | *E. coli* F- *mcrA* Δ(*mrr-hsd*RMS-*mcr*BC) Φ80*lac*ZΔM15 Δ *lac*X74 *rec*A1*ara*D139 Δ(*araleu*)7697 *gal*U *gal*K *rps*L (StrR) *end*A1 *nup*G | Invitrogen |
| MG1655 | *E. coli* K-12 F^–^ λ^–^ *ilvG*^–^ *rfb-50* *rph-1* | (81) |
| TJ-LM | *E. coli* commensal isolated from healthy NIH Swiss mouse | (15) |
| TJ-WM | *E. coli* commensal isolated from wild mouse feces | (15) |
| TJ-LR | *E. coli* commensal isolated from laboratory rat | (15) |
| MC4100 | *E. coli* F^-^ [araD139]_B/r_ Δ(argF-lac)169 &lambda^-^ e14- flhD5301 Δ(fruK-yeiR)725 (fruA25) relA1 rpsL150(strR) rbsR22 Δ(fimB-fimE)632(::IS*1*) *deoC1* | (82) |
| W3110 | *E. coli* F^-^ λ^-^ rph-1 INV(rrnD, rrnE) | (83) |
| BW25113 | *E. coli* lacI^q^ rrnB_T14_ ΔlacZ_WJ16_ hsdR514 ΔaraBA- D_AH33_ ΔrhaBAD_LD78_ | (76) |
| J96 | Uropathogenic *E. coli* isolated from pyelonephritis patient | (16) |
| E2348/69 | Prototypical enteropathogenic *E. coli* O127:H6 | (17) |
| Nissile 1917 | Commensal *E. coli* isolated from feces of healthy soldier | (18) |
| DBS100 | *C. rodentium* | (84) |
| MFDpir | MG1655 RP4-2-Tc::[ΔMu1::aac(3)IV-ΔaphA-Δnic35-ΔMu2::zeo] ΔdapA::(erm-pir) ΔrecA | (46) |
