# Supplementary material for: Isolation and Characterization of a Novel Temperate Escherichia coli Bacteriophage, Kapi1, Which Modifies the O-Antigen and Contributes to the Competitiveness of Its Host during Colonization of the Murine Gastrointestinal Tract
Source: mBio. 2022 Jan 25;13(1):e02085-21. doi: 10.1128/mbio.02085-21 (PMC8787464; doi:10.1128/mbio.02085-21)
Supplement: TABLE S2 [file mbio.02085-21-st002.docx]

**Table S2. Nucleotide primers used in this study.**

| Primer name | Sequence (5’ – 3’) | Source/Reference |
| --- | --- | --- |
| *nuoA* F  *nuoA* R | CAA TCA CCA GAG ATT CAC GC  CAT CAG GCA ACA CAG GCC | This study |
| Intact_1 F  Intact_1 R | CAT GGG TAA AAA TCC GGT GG  TCA GTT CGC TCC CAT CCG | This study |
| Incomplete_1 F  Incomplete_1 R | GAA CAT AGA AGC AGG ACT ACC  GGT CTT CTG GGC TGA TCT G | This study |
| Incomplete_2 F  Incomplete_2 R | CGA TTC GTG CAC GAT AGT G  CGA AAC CGG AAT GAT GAC C | This study |
| Incomplete_3 F  Incomplete_3 R | GAC AGG AAA GTC CTG CTC  GTT GCA ATG CAT CAG CTG | This study |
| Questionable_1 F  Questionable_1 R | CAC CAT GAA GCG TTT CAC  CTC AAT CTC TTC ACC GGA TGA | This study |
| Questionable_2 F  Questionable_2 R | GCT CCG TCA GAA AGA CC  GTT CTG TGC GGA TCA CTT TC | This study |
| Chromosome_left  Prophage_left | GGA AGG AAA CGT CTG GTA C  GCA CAT GCA GAC GTA ACC | This study |
| Chromosome_right  Prophage_right | GAGT CAT GCA CAG ATT CG  GCA TTG TTT CCT CCA CAG G | This study |
