# Supplementary material for: Isolation and Characterization of a Novel Temperate Escherichia coli Bacteriophage, Kapi1, Which Modifies the O-Antigen and Contributes to the Competitiveness of Its Host during Colonization of the Murine Gastrointestinal Tract
Source: mBio. 2022 Jan 25;13(1):e02085-21. doi: 10.1128/mbio.02085-21 (PMC8787464; doi:10.1128/mbio.02085-21)
Supplement: TABLE S3 [file mbio.02085-21-st003.docx]

**Table S3. Genome annotation of bacteriophage Kapi1.**

| **CDS** | **Coding region** | **Length (aa)** | **Strand** | **Start codon** | **Putative function** | **BLASTp hit** | **Query coverage** | **E-value** | **Identity** | **Accession** |
| --- | --- | --- | --- | --- | --- | --- | --- | --- | --- | --- |
| attP | 1-17 |  |  |  | phage attachment site |  |  |  |  |  |
| xis | 91-291 | 67 | (-) | ATG | excisionase | putative excisionase [Klebsiella phage vB_Kpn_Chronis] | 98% | 2E-38 | 86.36% | QEG04427.1 |
| hyp1 | 349-516 | 56 | (-) | ATG | hypothetical protein | hypothetical protein ECRS218_0063 [Enterobacteria phage CUS-3] | 98% | 5E-35 | 100% | ABQ88443.1 |
| hyp2 | 552-863 | 104 | (-) | ATG | hypothetical protein | hypothetical protein [Enterobacteria phage H19J] | 99% | 5E-72 | 99.03% | CAB38713.1 |
| hyp3 | 1009-1287 | 93 | (-) | ATG | hypothetical protein | hypothetical protein mEp043_031 [Enterobacteria phage mEp043 c-1] | 98% | 5E-64 | 100% | YP_007111531.1 |
| eaa | 1287-1934 | 216 | (-) | ATG | Eaa protein | Eaa prophage protein [Escherichia phage phiv142-3] | 99% | 6E-112 | 88.89% | [QGF19638.1](https://www.ncbi.nlm.nih.gov/protein/QGF19638.1?report=genbank&log$=prottop&blast_rank=1&RID=72P8TJB201N) |
| hyp4 | 1937-2128 | 64 | (-) | ATG | hypothetical protein | hypothetical protein [Escherichia phage TL-2011a] | 98% | 1E-36 | 96.83% | [AEW24523.1](https://www.ncbi.nlm.nih.gov/protein/AEW24523.1?report=genbank&log$=prottop&blast_rank=1&RID=72PH2R1A014) |
| hyp5* | 2130-2621 | 164 | (-) | ATG | hypothetical protein | putative acyltransferase [uncultured Mediterranean phage uvMED] | 68% | 6E-38 | 57.14% | [BAR19615.1](https://www.ncbi.nlm.nih.gov/protein/BAR19615.1?report=genbank&log$=prottop&blast_rank=4&RID=72PKU1SG014) |
| ead/  ea22 | 2630-2995 | 122 | (-) | ATG | Ead/Ea22-like protein | EaA protein [Salmonella phage SE16] | 68% | 3E-46 | 91.57% | [QDH45186.1](https://www.ncbi.nlm.nih.gov/protein/QDH45186.1?report=genbank&log$=prottop&blast_rank=1&RID=72PTZ450016) |
| hyp6 | 2992-3156 | 55 | (-) | ATG | hypothetical protein | hypothetical protein HK620p09 [Salmonella virus HK620] | 98% | 6E-32 | 98.15% | [NP_112042.1](https://www.ncbi.nlm.nih.gov/protein/NP_112042.1?report=genbank&log$=prottop&blast_rank=1&RID=72R0D6NM016) |
| hyp7 | 3173-3487 | 105 | (-) | ATG | hypothetical protein | hypothetical protein mEpX1_032 [Escherichia phage mEpX1_032] | 99% | 1E-68 | 99.04% | [YP_007111669.1](https://www.ncbi.nlm.nih.gov/protein/YP_007111669.1?report=genbank&log$=prottop&blast_rank=1&RID=72R2WBNE014) |
| gp157 | 3499-3981 | 161 | (-) | GTG | Gp157-like protein | Siphovirus GP157 family protein [Enterobacteriaceae] | 99% | 2E-108 | 99.38% | WP_000041326.1 |
| rec | 3965-4876 | 304 | (-) | ATG | RecT family recombinase | RecT family recombination protein [Escherichia phage mEpX1] | 99% | 0 | 99.34% | [YP_007111671.1](https://www.ncbi.nlm.nih.gov/protein/YP_007111671.1?report=genbank&log$=prottop&blast_rank=1&RID=74PA26P6014) |
| hyp8 | 4873-5181 | 103 | (-) | ATG | hypothetical protein | DnaB-like DNA helicase [Virbio phage 1.210.O._10N.222.52.C2] | 48% | 8.8 | 27.12% | [AUR95690.1](https://www.ncbi.nlm.nih.gov/protein/AUR95690.1?report=genbank&log$=prottop&blast_rank=6&RID=74PFHRR8014) |
| hyp9 | 5162-5269 | 36 | (-) | ATG | hypothetical protein | hypothetical protein [Salmonella phgae 118970_sal4] | 77% | 4E-10 | 85.71% | [YP_009274656.1](https://www.ncbi.nlm.nih.gov/protein/YP_009274656.1?report=genbank&log$=prottop&blast_rank=1&RID=74PMK0HK016) |
| kil | 5266-5418 | 51 | (-) | ATG | kil protein | kil protein [Escherichia virus HK97] | 98% | 2E-29 | 100.00% | [NP_037729.1](https://www.ncbi.nlm.nih.gov/protein/NP_037729.1?report=genbank&log$=prottop&blast_rank=1&RID=74PP6UUD014) |
| CIII | 5403-5588 | 62 | (-) | ATG | CIII regulatory protein | CIII protein [Escherichia phage mEpX2] | 98% | 4E-38 | 100.00% | [YP_007111473.1](https://www.ncbi.nlm.nih.gov/protein/YP_007111473.1?report=genbank&log$=prottop&blast_rank=1&RID=74PW6Y1C014) |
| hyp10 | 5621-5869 | 83 | (-) | ATG | hypothetical protein | hypothetical protein HK446_036 [Escherichia phage HK446] | 98% | 3E-52 | 100.00% | [YP_007111989.1](https://www.ncbi.nlm.nih.gov/protein/YP_007111989.1?report=genbank&log$=prottop&blast_rank=1&RID=74R1JAMD014) |
| hyp11 | 6044-6727 | 228 | (-) | TTG | hypothetical protein | hypothetical protein mEp235_037 [Enterobacteria phage mEp235] | 90% | 2E-142 | 94.69% | [YP_007111613.1](https://www.ncbi.nlm.nih.gov/protein/YP_007111613.1?report=genbank&log$=prottop&blast_rank=1&RID=74R3HDA5014) |
| nun | 6804-7142 | 113 | (-) | ATG | nun transcriptional terminator | transcription termination factor Nun [Enterobacteria phage mEp235] | 99% | 2E-77 | 98.21% | [YP_007111614.1](https://www.ncbi.nlm.nih.gov/protein/YP_007111614.1?report=genbank&log$=prottop&blast_rank=2&RID=74RCBK3K016) |
| N | 7145-7450 | 102 | (-) | ATG | N antitermination protein | antitermination protein N [Escherichia phage mEpX1] | 99% | 2E-65 | 98.02% | [YP_007111677.1](https://www.ncbi.nlm.nih.gov/protein/YP_007111677.1?report=genbank&log$=prottop&blast_rank=1&RID=74RF4SR7014) |
| CI | 7765-8415 | 217 | (-) | ATG | CI transcriptional repressor protein | prophage repressor [Enterobacteria phage mEp235] | 99% | 1E-160 | 100.00% | [YP_007111616.1](https://www.ncbi.nlm.nih.gov/protein/YP_007111616.1?report=genbank&log$=prottop&blast_rank=1&RID=74RGRH6Y014) |
| cro | 8496-8681 | 62 | (+) | ATG | Cro transcriptional repressor protein | prophage antirepressor [Enterobacteria phage mEp235] | 98% | 1E-37 | 100.00% | [YP_007111617.1](https://www.ncbi.nlm.nih.gov/protein/YP_007111617.1?report=genbank&log$=prottop&blast_rank=1&RID=74RNEFKT014) |
| CII | 8797-9093 | 99 | (+) | ATG | CII transcriptional activator protein | regulatory protein CII [Escherichia phage APC_JM3.2] | 98% | 7E-68 | 98.98% | [ATN92762.1](https://www.ncbi.nlm.nih.gov/protein/ATN92762.1?report=genbank&log$=prottop&blast_rank=1&RID=74RTWG8D014) |
| P | 9116-9388 | 91 | (+) | ATG | putative replication protein P | hypothetical protein [Escherichia phage TL-2011a] | 98% | 2E-59 | 98.89% | [AEW24537.1](https://www.ncbi.nlm.nih.gov/protein/AEW24537.1?report=genbank&log$=prottop&blast_rank=1&RID=74RWV72S016) |
| O | 9391-10338 | 316 | (+) | ATG | replication protein O | bacteriophage replication protein O [Escherichia phage TL-2011a] | 99% | 0 | 96.83% | [AEW24515.1](https://www.ncbi.nlm.nih.gov/protein/AEW24515.1?report=genbank&log$=prottop&blast_rank=3&RID=74S26SU0016) |
| dnaB | 10335-11711 | 459 | (+) | ATG | DnaB-like replicative helicase | replicative DNA helicase [Enterobacteria phage CUS-3] | 99% | 0 | 99.56% | [ABQ88420.1](https://www.ncbi.nlm.nih.gov/protein/ABQ88420.1?report=genbank&log$=prottop&blast_rank=2&RID=74U16V55014) |
| hyp12 | 11784-11990 | 69 | (+) | GTG | hypothetical protein | hypothetical protein HK022_58 [Escherichia virus HK022] | 98% | 1E-35 | 94.12% | [AUM59826.1](https://www.ncbi.nlm.nih.gov/protein/AUM59826.1?report=genbank&log$=prottop&blast_rank=1&RID=74U9898K016) |
| ninB | 11998-12408 | 137 | (+) | ATG | NinB protein | NinB [Escherichia phage phiv205-1] | 99% | 2E-87 | 100.00% | [QGF19942.1](https://www.ncbi.nlm.nih.gov/protein/QGF19942.1?report=genbank&log$=prottop&blast_rank=1&RID=74UBRPJ5016) |
| ninE | 12405-12581 | 59 | (+) | ATG | NinE protein | NinE protein [Enterobacteria phage HK106] | 98% | 2E-35 | 98.28% | [YP_007151724.1](https://www.ncbi.nlm.nih.gov/protein/YP_007151724.1?report=genbank&log$=prottop&blast_rank=2&RID=74UGD3VF016) |
| ninX | 12578-12958 | 127 | (+) | ATG | NinX protein | NinX [Salmonella phage vB_SenS_Sasha] | 92% | 3E-29 | 51.69% | [APU92816.1](https://www.ncbi.nlm.nih.gov/protein/APU92816.1?report=genbank&log$=prottop&blast_rank=2&RID=74UK76UH016) |
| ninF | 12951-13127 | 59 | (+) | ATG | NinF protein | NinF [Escherichia phage PA28] | 98% | 1E-35 | 98.28% | [YP_009601918.1](https://www.ncbi.nlm.nih.gov/protein/YP_009601918.1?report=genbank&log$=prottop&blast_rank=1&RID=74UNMYAP014) |
| roi | 13120-13842 | 241 | (+) | ATG | DNA binding protein | DNA-binding protein Roi [Stx2-converting phage 86] | 100% | 2E-176 | 99.58% | [YP_794124.1](https://www.ncbi.nlm.nih.gov/protein/YP_794124.1?report=genbank&log$=prottop&blast_rank=2&RID=7WFGAC5Z016) |
| nuc | 13842-14132 | 97 | (+) | ATG | endodeoxyribonuclease | endodeoxyribonuclease [Escherichia phage ECP1] | 97% | 3E-65 | 97.89% | [ASJ79465.1](https://www.ncbi.nlm.nih.gov/protein/ASJ79465.1?report=genbank&log$=prottop&blast_rank=7&RID=74V0DY7D016) |
| rusA | 14129-14491 | 121 | (+) | ATG | RusA-family holliday junction resolvase | Holliday-junction resolvase [Escherichia phage HK542] | 99% | 1E-81 | 99.17% | [YP_007151783.1](https://www.ncbi.nlm.nih.gov/protein/YP_007151783.1?report=genbank&log$=prottop&blast_rank=1&RID=74V3SJ8D016) |
| ninH | 14488-14676 | 63 | (+) | ATG | NinH protein | NinH protein [Escherichia phage HK629] | 96% | 4E-38 | 100.00% | [YP_007111834.1](https://www.ncbi.nlm.nih.gov/protein/YP_007111834.1?report=genbank&log$=prottop&blast_rank=2&RID=74VAV0W3014) |
| Q | 14673-15296 | 208 | (+) | ATG | Q antitermination protein | late gene regulator Q [Enterobacteria phage mEp235] | 99% | 1E-139 | 100.00% | [YP_007111631.1](https://www.ncbi.nlm.nih.gov/protein/YP_007111631.1?report=genbank&log$=prottop&blast_rank=1&RID=74VDM4KA014) |
| tRNA-Asn | 15571-15645 |  | (+) | ATG | tRNA-Asn |  |  |  |  |  |
| tRNA-Thr | 15651-15726 |  | (+) | ATG | tRNA-Thr |  |  |  |  |  |
| holin | 15969-16292 | 162 | (+) | ATG | holin | holin [Enterobacteria phage mEp235] | 99% | 4E-72 | 99.07% | [YP_007111632.1](https://www.ncbi.nlm.nih.gov/protein/YP_007111632.1?report=genbank&log$=prottop&blast_rank=2&RID=74VVUJZ1014) |
| lysin | 16276-16752 | 159 | (+) | ATG | glycoside hydrolase family lysozyme (lysin) | lysin [Escherichia virus HK97] | 99% | 4E-116 | 100.00% | [NP_037753.1](https://www.ncbi.nlm.nih.gov/protein/NP_037753.1?report=genbank&log$=prottop&blast_rank=1&RID=74VX3KKP014) |
| Rz | 16749-17216 | 156 | (+) | ATG | Rz endopeptidase | Rz lysis protein [Enterobacteria phage mEp460] | 99% | 4E-95 | 96.13% | [YP_007112131.1](https://www.ncbi.nlm.nih.gov/protein/YP_007112131.1?report=genbank&log$=prottop&blast_rank=4&RID=750247EM016) |
| hyp13 | 17213-17356 | 48 | (+) | ATG | hypothetical protein | hypothetical protein mEp043_068 [Enterobacteria phage mEp043 c-1] | 68% | 4E-16 | 96.97% | [YP_007111568.1](https://www.ncbi.nlm.nih.gov/protein/YP_007111568.1?report=genbank&log$=prottop&blast_rank=1&RID=750993NY016) |
| hyp14 | 17438-17959 | 174 | (+) | ATG | hypothetical protein | hypothetical protein [Klebsiella phage ST512-KPC3phi13.3] | 99% | 3E-120 | 92.49% | [QBQ71862.1](https://www.ncbi.nlm.nih.gov/protein/QBQ71862.1?report=genbank&log$=prottop&blast_rank=1&RID=750C30C0016) |
| hyp15 | 18263-18505 | 81 | (+) | ATG | hypothetical protein | hypothetical protein HK620p40 [Salmonella virus HK620] | 98% | 3E-52 | 100.00% | [NP_112073.1](https://www.ncbi.nlm.nih.gov/protein/NP_112073.1?report=genbank&log$=prottop&blast_rank=1&RID=750FESZY014) |
| hyp16 | 18507-18686 | 60 | (+) | GTG | hypothetical protein | hypothetical protein Sf101_0066 [Enterobacteria phage Sf101] | 98% | 8E-35 | 98.31% | [YP_009153140.1](https://www.ncbi.nlm.nih.gov/protein/YP_009153140.1?report=genbank&log$=prottop&blast_rank=1&RID=750J6NTK014) |
| terS | 18704-19132 | 143 | (+) | ATG | terminase small subunit | terminase small subunit [Salmonella virus HK620] | 97% | 2E-100 | 100.00% | [NP_112075.1](https://www.ncbi.nlm.nih.gov/protein/NP_112075.1?report=genbank&log$=prottop&blast_rank=2&RID=750MMP1H016) |
| terL | 19129-20544 | 472 | (+) | ATG | terminase large subunit | terminase large subunit [Enterobacteria phage CUS-3] | 99% | 0 | 99.79% | [ABQ88401.1](https://www.ncbi.nlm.nih.gov/protein/ABQ88401.1?report=genbank&log$=prottop&blast_rank=1&RID=750PMDUS014) |
| portal | 20546-22744 | 733 | (+) | ATG | portal protein | phage portal protein [Enterobacteria phage Sf101] | 99% | 0 | 99.45% | [YP_009153077.1](https://www.ncbi.nlm.nih.gov/protein/YP_009153077.1?report=genbank&log$=prottop&blast_rank=1&RID=750UR472014) |
| scaffold | 22835-23728 | 298 | (+) | ATG | scaffold protein | scaffold protein [Enterobacteria phage Sf101] | 99% | 0 | 98.32% | [YP_009153078.1](https://www.ncbi.nlm.nih.gov/protein/YP_009153078.1?report=genbank&log$=prottop&blast_rank=1&RID=750Z7GHJ016) |
| coat | 23747-25000 | 298 | (+) | ATG | coat protein | coat protein [Enterobacteria phage Sf101] | 99% | 0 | 98.56% | [YP_009153079.1](https://www.ncbi.nlm.nih.gov/protein/YP_009153079.1?report=genbank&log$=prottop&blast_rank=1&RID=7512R1U9016) |
| hyp17 | 25003-25230 | 76 | (+) | TTG | hypothetical protein | hypothetical protein Sf101_0006 [Enterobacteria phage Sf101] | 81% | 2E-39 | 100.00% | [YP_009153080.1](https://www.ncbi.nlm.nih.gov/protein/YP_009153080.1?report=genbank&log$=prottop&blast_rank=1&RID=751U7GHN014) |
| dsp1 | 25211-25672 | 154 | (+) | ATG | DNA stabilization protein | DNA stabilization protein [Escherichia phage phiv205-1 | 100% | 1E-108 | 98.69% | [QGF19920.1](https://www.ncbi.nlm.nih.gov/protein/QGF19920.1?report=genbank&log$=prottop&blast_rank=2&RID=7WFGAC5Z016) |
| dsp2 | 25682-27100 | 473 | (+) | ATG | DNA stabilization protein | DNA stabilization protein [Enterobacteria phage Sf101] | 99% | 0 | 99.58% | [YP_009153082.1](https://www.ncbi.nlm.nih.gov/protein/YP_009153082.1?report=genbank&log$=prottop&blast_rank=2&RID=75233S8W014) |
| tkp | 27100-27948 | 283 | (+) | ATG | tail needle knob protein | tail accessory protein [Salmonella phage UPF_BP1] | 48% | 9E-52 | 62.77% | [AOZ63903.1](https://www.ncbi.nlm.nih.gov/protein/AOZ63903.1?report=genbank&log$=prottop&blast_rank=6&RID=7528A8S7016) |
| hyp18 | 27948-28427 | 160 | (+) | ATG | hypothetical protein | putative head assembly protein [Enterobacteria phage CUS-3] | 71% | 0.065 | 22.03% | [ABQ88393.1](https://www.ncbi.nlm.nih.gov/protein/ABQ88393.1?report=genbank&log$=prottop&blast_rank=6&RID=752FN322014) |
| dtp | 28402-29076 | 225 | (+) | ATG | DNA transfer protein | DNA transfer protein [Enterobacteria phage IME10] | 64% | 9E-34 | 69.08% | [YP_007004329.1](https://www.ncbi.nlm.nih.gov/protein/YP_007004329.1?report=genbank&log$=prottop&blast_rank=2&RID=752KH9WR016) |
| dep | 29086-30429 | 448 | (+) | ATG | DNA ejection protein | DNA transfer protein [Salmonella phage SEN22] | 99% | 0 | 92.39% | [YP_009191466.1](https://www.ncbi.nlm.nih.gov/protein/YP_009191466.1?report=genbank&log$=prottop&blast_rank=1&RID=752T9340014) |
| hyp19* | 30414-32582 | 723 | (+) | ATG | hypothetical protein | putative lytic transglycosylase [Escherichia phage Sortsne] | 66% | 6E-48 | 29.17% | [QBZ71595.1](https://www.ncbi.nlm.nih.gov/protein/QBZ71595.1?report=genbank&log$=prottop&blast_rank=3&RID=752WTPAC014) |
| hyp20 | 32628-32936 | 103 | (-) | GTG | hypothetical protein | hypothetical protein BCF76_00950 [Escherichia coli] | 99% | 8E-69 | 100.00% | OCJ87778.1 |
| hyp21 | 32988-33170 | 61 | (-) | ATG | hypothetical protein | hypothetical protein [Escherichia coli] | 98% | 4E-26 | 100.00% | WP_139093999.1 |
| hyp22 | 33109-33387 | 62 | (+) | ATG | hypothetical protein | hypothetical protein [Salmonella phage vB_SemP_Emek] | 98% | 3E-36 | 100.00% | [YP_006560567.1](https://www.ncbi.nlm.nih.gov/protein/YP_006560567.1?report=genbank&log$=prottop&blast_rank=1&RID=753KMVH5016) |
| hyp23 | 33362-33571 | 70 | (-) | ATG | hypothetical protein | hypothetical protein [Salmonella phage vB_SemP_Emek] | 98% | 2E-28 | 98.55% | [YP_006560568.1](https://www.ncbi.nlm.nih.gov/protein/YP_006560568.1?report=genbank&log$=prottop&blast_rank=1&RID=753NCMFC016) |
| arc1 | 33568-33804 | 79 | (-) | ATG | Arc-like transcriptional regulator | Arc family DNA binding protein [Klebsiella phage ST899-OXA48phi17.1] | 98% | 4E-37 | 79.49% | [QBP28570.1](https://www.ncbi.nlm.nih.gov/protein/QBP28570.1?report=genbank&log$=prottop&blast_rank=1&RID=753PR6Y9014) |
| arc2 | 33837-34001 | 55 | (+) | TTG | Arc-like transcriptional regulator | Arc family DNA binding protein [Klebsiella phage ST899-OXA48phi17.1] | 81% | 1E-09 | 71.11% | [QBP28571.1](https://www.ncbi.nlm.nih.gov/protein/QBP28571.1?report=genbank&log$=prottop&blast_rank=1&RID=753UWPGW01R) |
| ant | 33998-34942 | 315 | (+) | TTG | Ant-like transcriptional antiterminator | antirepressor [Salmonella phage vB_SemP_Emek] | 69% | 2E-125 | 78.95% | [YP_006560573.1](https://www.ncbi.nlm.nih.gov/protein/YP_006560573.1?report=genbank&log$=prottop&blast_rank=1&RID=753WXHFM01R) |
| HNH | 35053-35553 | 167 | (-) | ATG | HNH homing endonuclease | HNH homing endonuclease [Salmonella phage vB_SenM_SB18] | 83% | 7E-34 | 46.15% | [QHI00658.1](https://www.ncbi.nlm.nih.gov/protein/QHI00658.1?report=genbank&log$=prottop&blast_rank=3&RID=7542JK6B016) |
| tsp | 35576-37951 | 792 | (+) | ATG | tail spike protein | tail protein [Escherichia phage N7] | 21% | 5E-60 | 67.43% | [AYR02211.1](https://www.ncbi.nlm.nih.gov/protein/AYR02211.1?report=genbank&log$=prottop&blast_rank=1&RID=7545J47E014) |
| hyp24* | 37982-38086 | 35 | (-) | ATG | hypothetical protein | hypothetical protein LCMAC102_0090 [Marseillevirus LCMAC102] | 29 | 4.2 | 45 | QBK86304.1 |
| int | 38123-39277 | 385 | (-) | ATG | integrase | integrase [Enterobacteria phage CUS-3] | 99% | 0 | 99.48% | [ABQ88382.1](https://www.ncbi.nlm.nih.gov/protein/ABQ88382.1?report=genbank&log$=prottop&blast_rank=1&RID=754A6HZC014) |
| hyp25* | 39276-39434 | 53 | (+) | ATG | hypothetical protein | hypothetical protein HK633_026 [Escherichia phage HK633] | 88 | 5E-22 | 85 | YP_007112301.1 |

* putative O-antigen modification genes
